# Ibrutinib modifies the function of monocyte/macrophage population in chronic lymphocytic leukemia

**DOI:** 10.18632/oncotarget.11782

**Published:** 2016-09-01

**Authors:** Stefania Fiorcari, Rossana Maffei, Valentina Audrito, Silvia Martinelli, Elisa ten Hacken, Patrizia Zucchini, Giulia Grisendi, Leonardo Potenza, Mario Luppi, Jan A. Burger, Silvia Deaglio, Roberto Marasca

**Affiliations:** ^1^ Hematology Unit, Department of Medical and Surgical Sciences, University of Modena and Reggio Emilia, Modena, Italy; ^2^ Department of Medical Sciences, University of Turin and Human Genetics Foundation, Turin, Italy; ^3^ Department of Leukemia, The University of Texas MD Anderson Cancer Center, Houston, TX, USA

**Keywords:** CLL, ibrutinib, microenvironment, nurse-like cells, immune modulation

## Abstract

In lymphoid organs, nurse-like cells (NLCs) show properties of tumor-associated macrophages, playing a crucial role in chronic lymphocytic leukemia (CLL) cell survival. Ibrutinib, a potent inhibitor of Bruton's tyrosine kinase (BTK), is able to counteract pro-survival signals in CLL cells. Since the effects on CLL cells have been studied in the last years, less is known about the influence of ibrutinib on NLCs properties. We sought to determine how ibrutinib modifies NLCs functions focusing on the balance between immunosuppressive and inflammatory features. Our data show that ibrutinib targets BTK expressed by NLCs modifying their phenotype and function. Treatment with ibrutinib reduces the phagocytic ability and increases the immunosuppressive profile of NLCs exacerbating the expression of M2 markers. Accordingly, ibrutinib hampers LPS-mediated signaling, decreasing STAT1 phosphorylation, while allows IL-4-mediated STAT6 phosphorylation. In addition, NLCs treated with ibrutinib are able to protect CLL cells from drug-induced apoptosis partially through the secretion of IL-10. Results from patient samples obtained prior and after 1 month of treatment with ibrutinib show an accentuation of CD206, CD11b and Tie2 in the monocytic population in the peripheral blood. Our study provides new insights into the immunomodulatory action of ibrutinib on monocyte/macrophage population in CLL.

## INTRODUCTION

Chronic lymphocytic leukemia (CLL) is the most common hematologic malignancy in the Western Countries. Despite several and important recent therapeutic advances, CLL is still an incurable disease. CLL is characterized by several clinical complications related to alterations in the immune system, including hypogammaglobulinemia, predisposition to infections and increased incidence of autoimmune disorders [1]. In this scenario, CLL cells are not innocent bystanders, but escape immunosurveillance and actively model the surrounding microenvironment to aberrantly orchestrate the function of immune effector cells [2]. In lymphatic tissues, CLL cells form areas of larger proliferating cells, known as pseudofollicles, establishing intimate contact with accessory cells [3].

Nurse-like cells (NLCs) are round or fibroblast-shaped adherent cells differentiated from peripheral blood-derived monocytes in vitro by high-mobility group protein B-1 (HMGB1) release from CLL cells and also detected in lymph nodes (LN) of CLL patients [4]. NLCs assume a pivotal role in leukemic clone maintenance supporting CLL survival, proliferation and protecting CLL from drug-induced apoptosis [5]. Accordingly, macrophage depletion was recently demonstrated as a promising strategy to sensitize CLL cells to apoptosis and inhibit disease progression in mouse model [6, 7]. Phenotypically, NLCs are considered as CLL-specific M2-skewed tumor associated macrophages characterized by high CD11b, CD163, CD206, HLA-DR, HGF and IDO expression [8–12] and by dysregulation of genes involved in immunocompetence [13]. Likewise, NLCs were reported to promote T regulatory cell expansion and to sustain T cell immune dysfunctions in CLL [6, 12].

Ibrutinib is a potent irreversible inhibitor of Bruton's tyrosin kinase (BTK) that has demonstrated exceptional safety and efficacy as a monotherapy in CLL patients [14, 15]. While durable clinical responses to single-agent ibrutinib are common in CLL, few patients experience a complete response. As consequence, in an effort to improve the already impressive effects of ibrutinib, it is currently investigated in combination with chemotherapy and monoclonal antibodies to facilitate the clearing of CLL cells and achieve deeper responses [16, 17]. Ibrutinib has been demonstrated to disrupt B-cell receptor (BCR) and NF-ĸB signaling pathways [18, 19], affecting lymphocytes migration and adhesion and counteracting the pro-survival signals derived from the microenvironment [20, 21]. However, ibrutinib is also a potent and clinically relevant immunomodulatory drug. Ibrutinib irreversibly binds IL-2 inducible T cell kinase (ITK) leading to impairment of NK function with decreased ADCC [22] and to inhibition of Th2 cells activation upon TCR stimulation [23]. In treated patients, ibrutinib was associated with a normalization of CD3+CD4+ and CD3+CD8+ T cell count and Th17 cell count, a decrease in T cell activation, proliferation and “pseudo-exhaustion” and a reduction of several inflammatory cytokines [23]. Overall, ibrutinib seems to correct the CLL-mediated chronic and dysfunctional activation of T cells, thereby enhancing immunotherapeutic strategies such as CAR-T cell therapy [24]. BTK is also expressed by macrophages and is required for phagocytosis and for macrophage lineage commitment to inflammatory profile [25]. Ibrutinib was reported to counteract the phagocytosis of rituximab-coated CLL cells by macrophages [26]. In ibrutinib-treated patients, NLCs can be generated in vitro and maintain their ability to rescue CLL cells from apoptosis [27]. Conversely, ibrutinib was found to disaggregate the interactions of macrophages and CLL cells in bone marrow microenvironment in treated patients [28].

These evidences prompted us to investigate the biological effects and mechanism mediated by ibrutinib on NLCs in CLL. Specifically, we sought to determine how ibrutinib modifies NLCs functions, focusing on the phagocytic activity and the balance between immunosuppressive and inflammatory features, and what are the consequences of these effects in the CLL-NLCs crosstalk. We demonstrate that treatment with ibrutinib targets BTK in NLCs, impairs phagocytosis and improves immunosuppressive phenotype. Overall, our study provides new insights into the effect of ibrutinib treatment on the modulation of immune elements in CLL tissue microenvironment, highlighting new mechanisms that may impair CLL sensitivity to ibrutinib.

## RESULTS

### BTK protein is expressed and targeted in NLCs by ibrutinib

**Figure 1:**
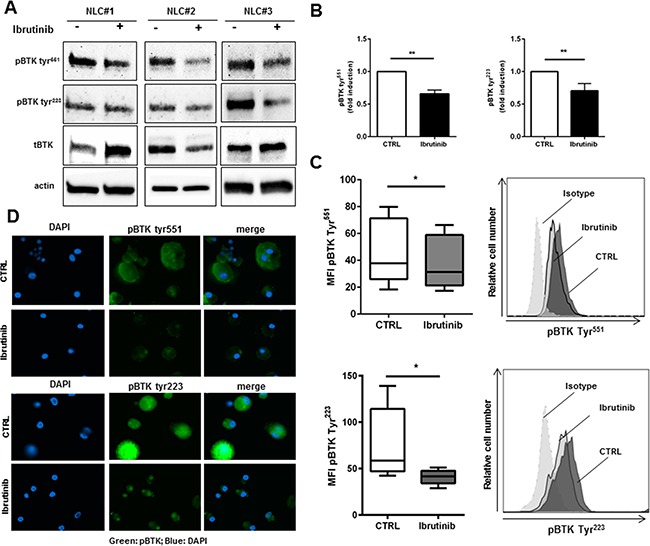
Ibrutinib specifically targets BTK in NLCs NLCs (n=5) were treated or not with ibrutinib 1μM for 1h. Then, cell lysates were analyzed by immunoblotting using anti-phosphoBTK Tyr^551^, Tyr^223^, total BTK and anti-actin antibodies. Three representative samples are depicted in panel **A**. In panel B bar diagram represents densitometric quantification of bands relative to phospho-BTK Tyr^551^ and phospho-BTK Tyr^223^ normalized on β-actin. Data are presented as mean ± SEM of 5 different NLCs samples (**P<0.01). NLCs were treated with ibrutinib for 1h before assessing expression of phospho-BTK Tyr^551^ and Tyr^223^ by flow cytometry **C.** and immunofluorescence microscopy **D.** (n=5, *P<0.05).

Since the expression of BTK is not restricted to B cells, but it is also present in myeloid cells like monocytes/macrophages [29], we sought to determine the expression profile and the activation status of BTK in NLCs. CLL cells were completely removed with several washes and after 1 hour of incubation with ibrutinib NLCs were collected. The purity of NLCs preparations was assessed by phase-contrast microscopy and by western blot with CD19 antibody as shown in Supplementary Figure S1. As shown in Figure 1, NLCs revealed the expression of BTK with two different sites of phosphorylation (Tyr^551^ and Tyr^223^). We asked ourselves whether ibrutinib would interfere with BTK activation in these cells. NLCs were treated with ibrutinib 1 μM for 1 hour and BTK phosphorylation was determined. Ibrutinib reduced the level of phosphorylated BTK at both sites Tyr^551^ and Tyr^223^ as determined by western blot (Figure 1A and Figure 1B n=5). This result was also confirmed by flow cytometry (Figure 1C) and immunofluorescence microscopy (Figure 1D). Moreover, treatment with ibrutinib affected the activation of BTK-downstream signaling pathways in NLCs as the PI3K and MAPK pathways leading to decreased AKT and ERK1/2 phosphorylation (Supplementary Figure S1). These results imply that ibrutinib, targeting BTK in NLCs, may modify the biological functions of NLCs. As consequence, in the following experiments we explore the effects of BTK inhibition on pivotal mechanisms of macrophage functionality such as phagocytic capacity and M1 vs. M2 polarization.

### Ibrutinib affects phagocytic activity of NLCs

**Figure 2:**
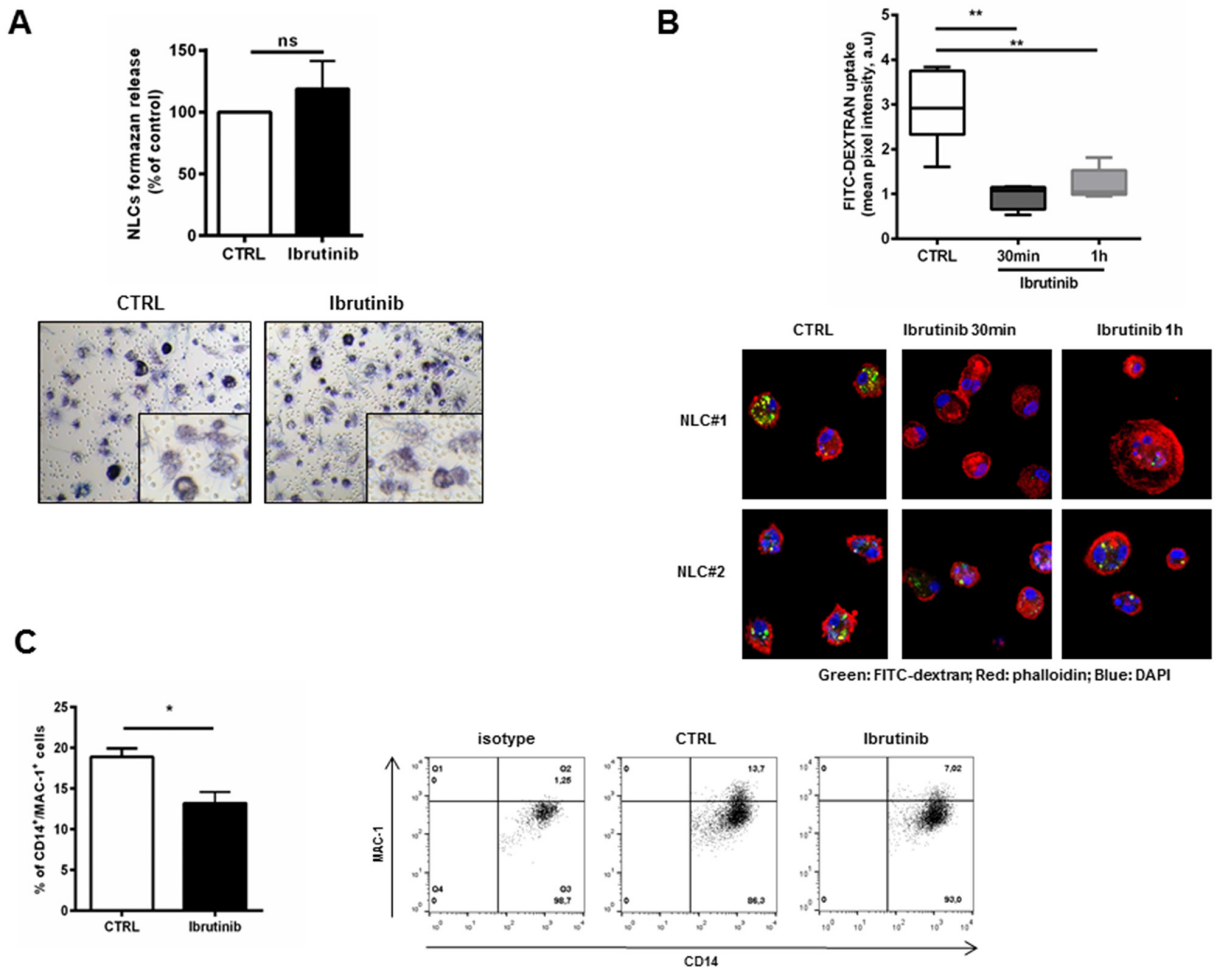
Ibrutinib impairs the phaghocytic activity of NLCs **A.** Bar diagram represents the formazan release by metabolically active NLCs treated with ibrutinib 1 μM for 24 hours compared to untreated control (n=6, ns). In the bottom panels, representative phase-contrast micrographs demonstrate NLCs activation (as insoluble formazan precipitate) after treatment with ibrutinib. **B.** Box-plots summarize FITC-Dextran uptake by NLCs treated or not with ibrutinib 1 μM relative to 6 independent experiments (**P<0.01). In the bottom panels, two representative NLCs samples show confocal staining of NLCs with FITC-dextran, phalloidin and DAPI. **C.** Bar diagram shows the percentage of positive CD14+ NLCs stained for MAC-1 Ab or isotype control (n=6, P<0.05). Contour plots show a representative sample.

We first observed that after ibrutinib treatment the activation status and viability of NLCs was not affected (Figure 2A) and the morphology of NLCs was preserved (Supplementary Figure S2). In macrophages, BTK is involved in cytoskeleton remodeling and is required for optimal phagocytosis in the process of ingestion and phagosomes formation [30]. In line with these evidences, we investigated the ability of NLCs to engulf and ingest particles after treatment with ibrutinib. We treated NLCs with ibrutinib for 30 minutes or 1 hour and uptake of FITC-dextran particles was quantified by confocal microscopy. Ibrutinib decreased NLCs phagocytic activity compared to control (n=6, P<0.01 for both time-points, Figure 2B). One of the main events accompanying phagocytic cup formation is the activation of MAC-1 (CD11b/CD18) [31]. Consistently, we also found an impairment of MAC-1 (CD11b/CD18) mean expression from 19% (±1%) to 13% (±1%) (n=6, P<0.05, Figure 2C).

### Ibrutinib enhances the immunosuppressive features of NLCs

**Figure 3:**
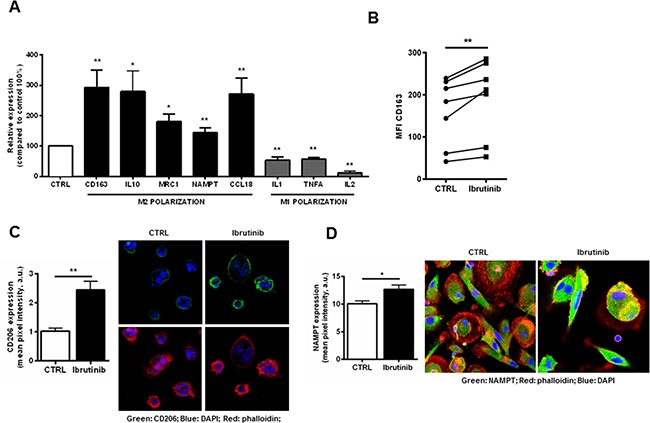
Exposure of NLCs to ibrutinib intensifies the expression of genes involved in M2 polarization **A.** NLCs from 8 CLL patients were exposed to ibrutinib or vehicle (DMSO) for 24 hours. Transcriptional levels of CD163, IL-10, MRC1, NAMPT, CCL18 and PD-L1 (M2 polarization) and IL-1, TNFα, IL-2 (M1 polarization) were measured by quantitative reverse-transcription PCR (n=8, *P<0.05, **P<0.01). **B.** Diagrams show CD163 fluorescence intensity of NLCs treated with ibrutinib for 24 hours. Values of untreated and treated samples (n=7) are connected by lines (P<0.01). **C.** Bar diagram shows cumulative analysis of CD206 pixel intensity scoring at least 10 different cells for 3 different samples (P<0.01). On the right one representative sample stained for CD206, DAPI and phalloidin. **D.** NAMPT expression on NLCs, treated with ibrutinib 1 μM for the indicated time, was evaluated by confocal microscopy (n=6) using anti-NAMPT and secondary Alexa-488-anti-rabbit antibodies. Phalloidin and DAPI were used to counterstain. Graph shows cumulative data of green fluorescence pixel intensity (n=6, P<0.05).

BTK is involved in macrophages lineage commitment to inflammatory profile [25]. Several evidences have shown that NLCs are closely associated to tumor-associated macrophages (TAM) with peculiar M2-skewed properties [8, 10, 11]. We sought to determine whether treatment with ibrutinib further stimulates the expression of M2 polarization markers. After 24 hours of treatment, the transcriptional signature of NLCs exposed to ibrutinib showed the induction of M2 markers CD163 (P<0.01), IL10 (P<0.05), MRC1 (CD206) (P<0.01), CCL18 (P<0.01) and PD-L1 (P<0.05) compared to control (n=8) and the concomitant down-regulation of M1 macrophages markers IL-1, TNFα and IL-2 (P<0.01 for all) (Figure 3A). These data were confirmed by an induction of the surface expression levels of M2 polarization markers CD163 and CD206 compared to untreated controls (n=7, P<0.01 for all) (Figure 3B and 3C). Of interest, we also detected the induction of NAMPT, known to enhance the immunosuppressive profile of NLCs [11] (Figure 3A and 3D).

### Ibrutinib stimulates M2 signaling pathways hampering a M1 profile in NLCs

**Figure 4:**
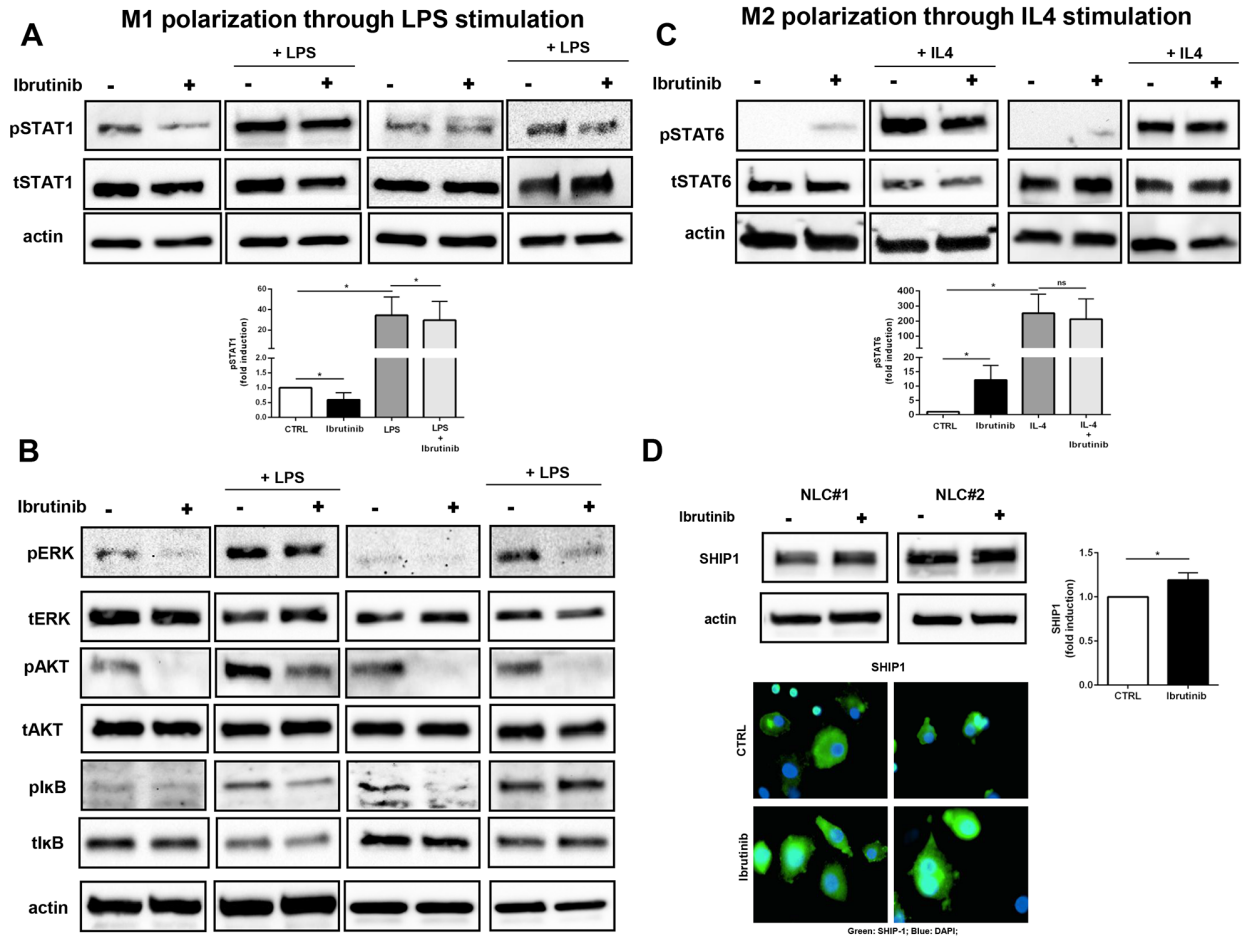
Ibrutinib supports M2 signaling pathways by interfering with M1 polarization NLCs were treated for 1 hour with ibrutinib 1 μM and then stimulated with LPS (100 ng/ml) or IL-4 (10 ng/ml) for 3 hours or 30 minutes respectively. Lysates were probed for pSTAT1, tSTAT1 **A.** pERK, tERK, pIĸB, tIĸB, pAKT, tAKT and actin **B.** following LPS stimulation investigating M1 polarization. **C.** PSTAT6, tSTAT6 and actin were determined after IL-4 stimulation for M2 polarization. **D.** NLCs were treated with ibrutinib and SHIP1 induction was monitored by western blot and by immunofluorescence. Bar diagram depicts densitometric quantification of bands relative to pSTAT1, pSTAT6 and SHIP1, either in presence or absence of the corresponding stimulus, normalized on β-actin. Data are presented as mean ± SEM of 5 different NLCs samples (*P<0.05).

M1 polarization is driven by IFNs and TLR signaling via STAT1, whereas M2 polarization is driven by IL-4 and IL-3 via STAT6 [32]. NLCs, treated for 1 hour with ibrutinib, displayed an impairment of STAT1 phosphorylation that was hampered also after LPS stimulation for 3 hours compared to the corresponding controls (Figure 4A and Supplementary Figure S3A). Additionally, ibrutinib affected the levels of other signaling pathways crucial for M1 commitment such as phosphorylation of ERK, AKT and IĸB either in presence or absence of LPS stimulation (Figure 4B). Consistent with an impairment of M1 signaling pathways, we also observed in ibrutinib-treated NLCs an induction of STAT6 phosphorylation, that was also preserved upon IL4 stimulation (Figure 4C and Supplementary Figure S3B). In addition, treatment with ibrutinib enhanced the expression of the anti-inflammatory protein SHIP1 (Figure 4D). These results indicate that inhibition of BTK in NLCs induced by ibrutinib intensifies the expression of M2-promoting properties counteracting the polarization toward M1 phenotype.

### IL-10 secreted by ibrutinib-treated NLCs exerts protective effects on CLL cells

**Figure 5:**
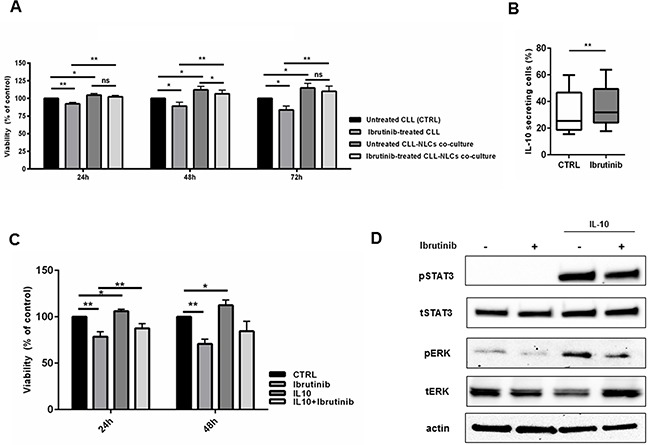
IL-10 mediates pro-survival signals in CLL cells during treatment with ibrutinib **A.** Viability of CLL cells were investigated treating both CLL cells and NLCs with ibrutinib 1 μM. CLL were harvested and divided into two fractions, one was placed back onto the autologous NLCs and the second was placed into wells without NLCs. Bar diagrams represent the mean relative CLL cell viabilities after 24, 48, 72h (n=9, *P<0.05, **P<0.01). **B.** Box plots show the percentage of IL-10 secreting cells in CD14+ NLCs population treated or not with ibrutinib for 1 hour relative to five independent experiments. **C.** Bar diagrams show the relative viability of CD19+ CLL cells treated with ibrutinib for 1 hour and then stimulated for 48 hours with IL-10 compared to untreated control (n=7, *P<0.05, **P<0.01). **D.** Western blot represents the activation of pro-survival signals induced by IL-10 either in presence or not of ibrutinib in one representative CD19+ CLL sample. Blots display pSTAT3, tSTAT3, pERK 1/2, tERK and actin.

Inside tissue microenvironments, NLCs establish a bidirectional cross-talk with CLL cells, allowing their protection from spontaneous and drug-induced apoptosis [5, 33]. We exposed both CLL cells and NLCs to ibrutinib. In presence of ibrutinib, CLL cells cultured without NLCs were more sensitive to apoptosis if compared to CLL cells cultured with NLCs. Indeed, as recently reported also by others [27], we found that ibrutinib does not decisively hamper the protective effect mediated by NLCs on leukemic cells when both CLL cells and NLCs were exposed to ibrutinib 1 μM (Figure 5A). This result means that NLCs maintain the ability to nurture CLL cells also in presence of ibrutinib, mediating signals that partially rescue CLL cells from drug-induced apoptosis. Given the importance of soluble factors on protecting CLL cells from apoptosis in the microenvironment, we next sought to investigate one of the possible cytokines secreted by ibrutinib-treated NLCs that may be involved in the maintenance of CLL cell survival. Because ibrutinib further stimulated the gene expression level of IL-10 in NLCs (Figure 3A), we explored its role in the cross-talk between NLCs and CLL cells. First, we demonstrated that ibrutinib was able to improve NLCs secretion of IL-10 (n=6, P<0.05) (Figure 5B). To better clarify the role of IL-10 in mediating CLL survival, we then cultured CD19^+^ CLL cells stimulated with IL-10 in a dose escalation experiment (from 0.1 ng/ml to 100 ng/ml) and we found a moderate protection from apoptosis at different doses (n=4, Supplementary Figure S4). We then analyzed the ability of ibrutinib to abrogate the pro-survival signal induced by IL-10 stimulation (1 ng/ml) in CLL cells after 24-48 hours of culture. As shown in Figure 5C, ibrutinib did not completely antagonize the ability of IL-10 to protect CLL cells from apoptosis (n=7) and to mediate signaling pathways through pSTAT3 and pERK (Figure 5D). Altogether these findings demonstrate an ibrutinib-mediated reinforcement of IL-10 production by NLCs and the inability of ibrutinib to totally counteract IL-10 signaling on CLL cells, suggesting that IL-10 may be involved in the mechanism of ibrutinib-resistance mediated by NLCs.

### Ibrutinib alters the monocytic population in treated CLL patients

**Figure 6:**
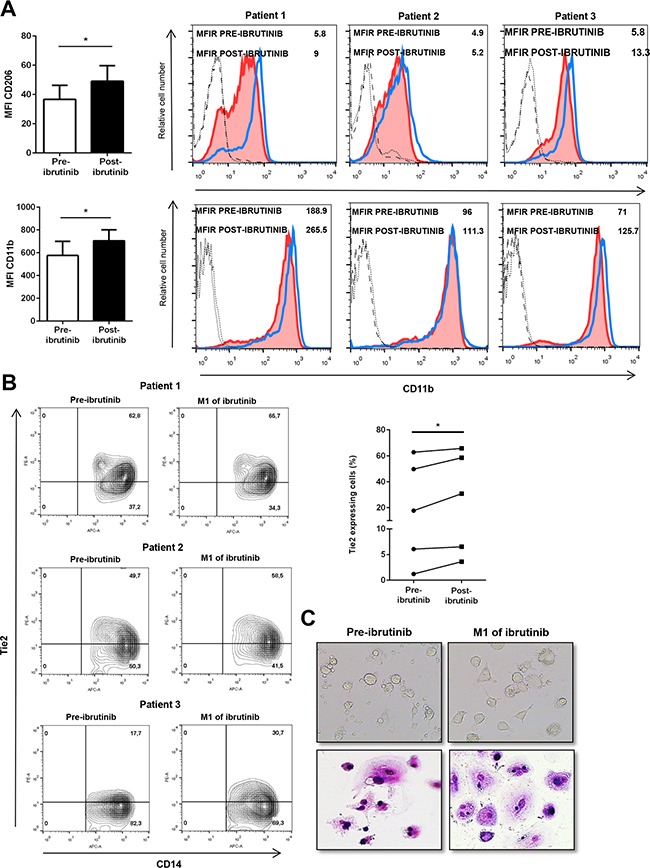
Ibrutinib alters the circulating monocytes in CLL patients **A.** Bar diagrams show cumulative analysis of CD206 (up) and CD11b (down) MFI for 5 different CLL patients in pre-treatment and after treatment samples. On the right, histograms show MFI of CD206 (up) and CD11b (down) for 3 representative CLL samples calculated using the corresponding isotype in pre-treated and after treatment samples. The blue histogram represents the post-treatment condition and the red shows the pre-treatment condition. **B.** Contour plots show the percentage of CD14+ Tie2+ monocytes before and after treatment with ibrutinib. On the right, diagram represents the percentage of positive CD14+ cells stained for Tie2 either before or after treatment with ibrutinib (n=5). **C.** Phase contrast photomicrographs and May-Grunwald Giemsa staining document the morphology of NLCs before and after treatment with ibrutinib.

To understand the *in vivo* alterations mediated by ibrutinib, we analyzed the circulating CD14+ population in peripheral blood samples of CLL patients before and after one month of treatment with ibrutinib. The blood monocytic population, selected with anti-CD14, displayed a higher positivity for CD206 after treatment with ibrutinib in all 5 CLL patients with an increase of MFI from 37 (±4) to 49 (±45) (Figure 6A, upper panel, *P<0.05). Again, we detected an induction of CD11b in the CD14+ monocytic population by ibrutinib from 576 (±55) to 705 (±473) (Figure 6A, lower panel, *P<0.05). Next, we evaluated the extent of a population of circulating monocytes able to express Tie2 receptor (TEM) and showing tumor-promoting properties [34]. In the CD14+ population, we found a variable expression of Tie2+ monocytes ranging from 3% to 62.8%, but in all patients analyzed we detected an increased expression after treatment with ibrutinib (Figure 6B, n=5, *P<0.05). Next, we cultured PBMCs from ibrutinib-treated patients to evaluate the capacity to generate NLCs. As shown in Figure 6C, ibrutinib did not prevent NLCs formation in vitro. In addition, NLCs viability as well as the capacity to support CLL cell survival were preserved (data not shown). Altogether, these results suggest that treatment with ibrutinib is able to induce modifications of the CD14+ population in CLL patients that may be evaluated in the complexity of the *in vivo* situation.

## DISCUSSION

Ibrutinib is a first-in-class potent inhibitor of BTK that binds covalently to Cys-481 in the ATP-binding domain of the kinase. Inhibition of BTK in CLL cells determines a disruption of important signaling pathways involved in survival, migration and adhesion of leukemic cells [19, 35]. This peculiar effect leads to a substantial delocalization of CLL cells from the protective tissue compartment to the periphery interfering with pathogenetic mechanisms of recirculation and homing. One question that needs to be answered is whether ibrutinib may affect the non-malignant cellular compartment by modifying the nurturing and protective niches of CLL cells into tissue microenvironments. Recent studies have demonstrated off-target effects of ibrutinib that could actively contribute to modulate the CLL microenvironment [22, 23, 26, 36]. Ibrutinib mainly targets ITK in T cells influencing Th1/Th2 polarization towards Th1 potentially modifying T cell anergy in CLL patients [23]. Moreover, impairment of NK function with decreased ADCC [22] and disruption of phagocytosis of rituximab-coated CLL cells by macrophages were related to ibrutinib treatment [26].

The ability of ibrutinib to effectively disrupt the crosstalk between CLL cells and NLCs is still unclear [18, 27, 28]. Our study provides new insights into the biological effects of ibrutinib treatment, reporting for the first time an extensive description of the molecular and functional modifications induced by ibrutinib treatment in NLCs. We demonstrate that ibrutinib is unable to completely antagonize the protective and nurturing role of NLCs allowing the protection of CLL cells from ibrutinib. This conclusion is in line with a recent report indicating that treatment with ibrutinib is not able to mobilize NLCs from niches into the blood and also to interfere with the pro-survival effects of NLCs [27]. Moreover, NLCs obtained *in vitro* from ibrutinib-treated patients are able to protect CLL cells as well as that of NLCs from untreated patients [27]. Here, we add a new explanation of this unwanted effect on NLCs. Ibrutinib targets BTK in NLCs affecting the activation by Src kinase-induced phosphorylation at Tyr^551^, autophosphorylation at Tyr^223^ [37] and the activation of downstream-related pathways. The relevance of BTK expression in macrophages is related to regulation of macrophage lineage commitment. In accordance, BTK is involved in the induction of signals driving M1 polarization, but it also functions as a negative regulator of M2-polarizing signaling pathways [25]. The BTK inhibition mediated by ibrutinib potentiates the M2-skewed features typical of NLCs. Specifically, ibrutinib induces (i) inhibition of phagocytosis in line with downregulation of MAC1 expression, (ii) up-regulation of CD163 and CD206 known to be M2 macrophage markers and (iii) modulation of a peculiar cluster of genes involved in immune suppression. Of interest, in the M2 signature, ibrutinib promotes the expression of NAMPT able to enhance the immunosuppressive phenotype of NLCs as well as their ability to protect CLL from apoptosis creating a CLL supporting microenvironment [11].

BTK is recruited to the Toll like receptor-4, during LPS stimulation, promoting phosphorylation of NFĸB and STAT1 activation, further sustaining the rationale of an involvement of BTK in M1 polarization [25]. When NLCs were exposed to LPS stimulation, STAT1 and its related signaling pathways were strongly activated. Treatment with ibrutinib, before LPS stimulation, diminished the activation of STAT1 arguing for a negative effect of this agent on M1 signaling pathway. On the contrary, the inhibition of BTK activity, promoted by ibrutinib, increased the induction of STAT6 also in presence of IL-4 as mediator of M2 polarization. Again, ibrutinib induced the expression of another M2 modulator, SHIP1, which is an anti-inflammatory protein that turns-off PI3K-dependent signaling [38].

CLL is partially rescued by ibrutinib-induced death by NLCs contact. We pointed our attention on IL-10, as a survival factor that may be involved in this effect given (i) ibrutinib intensely induces gene expression and secretion of IL-10 in NLCs, (ii) CLL cells express IL-10 receptor as previously demonstrated [39], (iii) elevated serum IL-10 levels are associated with worse CLL patients survival [40]. The role of IL-10 in mediating CLL survival is still controversial, implying a possible involvement in CLL cell maintenance or in cell death. In particular, IL-10 provokes apoptosis decreasing Bcl-2 protein levels [41] or activating STAT1 protein in CLL cells [42]. On the contrary, IL-10 enhances the survival of CLL cells acting as an autocrine growth factor [43, 44] and it is able to reduce cell death caused by hydrocortisone [39]. Here, IL-10 mediates pro-survival signals in CLL cells through the activation of STAT3, that is known to provide a survival advantage activating transcription of anti-apoptotic genes and ERK 1/2 signaling pathway [45]. Treatment with ibrutinib is not able to totally counteract the pro-survival effect of IL-10 in CLL cells arguing for its possible involvement in mediating resistance to ibrutinib.

Several studies demonstrate the considerable clinical success of ibrutinib showing a good safety profile and a promising clinical efficacy [14, 15]. Despite these exciting results, it had been immediately clear the limited capacity of this agent to induce a complete eradication of neoplastic clone, even if an improvement in quality of responses is seen when patients are treated for an extended period [46]. Persistent disease in blood and inside tissues can be detected in patients also after years of single-agent therapy. Moreover, some patients relapse during treatment and some patients fail to respond, sometimes developing a significant resistance to the treatment. Ibrutinib resistance is known in part to be related to acquired mutation in BTK at its binding site with a cysteine to serine mutation and others involving phospholipase Cγ2 (PLCγ2), an important downstream effector of BTK [47]. Nevertheless, the contribution of microenvironmental elements in CLL protection to ibrutinib may be envisioned and warrants further investigations in treated patients inside clinical trials. Our analysis of blood samples collected from 5 patients before and after one month of treatment with ibrutinib, illustrates modifications of the monocytic population in the peripheral blood. In ibrutinib-treated samples, we detected an increased expression of M2 markers as CD206 and CD11b and higher percentage of a small subpopulation of circulating monocytes able to express Tie2 receptor (TEM). The increased number of Tie2 expressing monocytes is somehow a dismal result, due to the peculiar pro-angiogenic activity and the tumor-promoting M2 phenotype of this monocytic subset.

Overall, our results are in line with the reported observation by Boissard and colleagues that NLCs may mediate ibrutinib resistance *in vitro*, but further provide a mechanistic explanation for these undesired effects [27]. On the other hand, a very recent analysis of CLL-associated macrophages in bone marrow biopsies obtained from CLL patients during ibrutinib treatment suggests that ibrutinib may disrupt CLL-macrophage interactions on the basis of a significant decrease in CD68+ cellular extension and a reduction of CXCL13 secretion [28]. It has to be considered that direct contact with CLL cells is essential for the supporting functions of NLCs. As consequence, a possible explanation of the apparent contradictory results may be that ibrutinib treatment in patients reduces the CLL infiltration inside bone marrow, probably interfering with the extent of CLL-NLCs contact and perhaps compensating for direct immunosuppressive effect of ibrutinib on NLCs. However, further evaluations of markers related to M1/M2 polarization in macrophages in bone marrow biopsies during ibrutinib treatment would be of interest. Ibrutinib is remarkably effective in CLL, but its mechanism of action inside the complexity of tumor microenvironment is not fully elucidated. For this reason, our results push for a more deep and accurate analysis of the immunomodulatory effects of ibrutinib to optimize its use and develop effective combination strategies. It could be of interest the observation that lenalidomide counteracts the pro-leukemia role and the immunodeficiency typical of NLCs inducing properties of pro-inflammatory cells [48]. The opposite effect exerted by lenalidomide and ibrutinib on NLCs is intriguing, suggesting future studies to understand whether lenalidomide may overcome the immunosuppressive effect on NLCs mediated by ibrutinib.

In conclusion, we proposed a new mechanism of action of ibrutinib that further miseducates NLCs, suggesting that this drug not only has an effect on the CLL clone but also extensively influences the cellular components of the CLL microenvironment. Ibrutinib supports the nurturing and protective behavior of NLCs potentiating their immunosuppressive profile and leading to secretion of unwanted survival factors. Our findings leave open the issue of how ibrutinib effects on CLL cells may be optimized by associating it with other agents in order to combine its peculiar mechanism of action related to disruption of survival signaling pathways, migration and adhesion, with an effective disruption of protective milieu in tissue microenvironment.

## MATERIALS AND METHODS

### Patients and samples

Blood samples from untreated patients that matched standard diagnostic criteria for CLL were obtained from the Hematology Unit of Modena Hospital, Italy with a protocol approved by the Institutional Review Board. All patients provided written informed consent in accordance with the declaration of Helsinki. Peripheral blood mononuclear cells (PBMCs) were isolated by Ficoll density gradient centrifugation and used fresh or cryopreserved in RPMI-1640 medium (Life Technologies, Carlsbad, CA, USA), 50% fetal bovine serum (FBS), and 10% dimethyl sulfoxide (DMSO) and stored in liquid nitrogen until use. To enrich for CLL cells, PBMCs were incubated with CD19 Microbeads (Miltenyi Biotec, Germany) obtain a purity > 99%, as assessed by flow cytometry. Ibrutinib was purchased from Selleckchem and was dissolved in DMSO, which was used as a vehicle control in all experiments. Ibrutinib was used at a dose of 1 μM as in previous studies [18, 20, 23].

### Nurse-like cells generation and analyses

PBMCs from CLL patients were suspended in RPMI with 10% FBS to a final concentration of 10^7^/ml, as previously published [5]. After 14 days, the non-adherent CLL cells were harvested vigorously pipetting the contents of the well leaving untouched the adherent cells. NLCs typical morphology and immunophenotype were confirmed by microscopy and flow cytometry, respectively.

### Cell treatments and viability

For co-culture with NLCs, PBMCs were suspended in medium to a concentration of 10^7^/ml and incubated for at least 10 days. To assess the impact of ibrutinib on CLL cell viability, both CLL and NLCs were concomitantly exposed to ibrutinib. In detail, CLL cells were removed by thoroughly pipetting and divided into 2 fractions. One was placed back onto the autologous NLCs and the second fraction was placed into wells without NLCs either. After 30 minutes, ibrutinib or DMSO was added in the corresponding wells. For all these experiments, ibrutinib was used at a dose of 1 μM. CLL cell viability was determined collecting non-adherent cells in the corresponding well and analyzed by flow cytometry using annexin V-FITC and propidium (PI) staining (eBioscience, San Diego, CA, USA) after 24h, 48h and 72h. For IL-10 stimulation, CD19^+^ CLL cells were pre-incubated in complete RPMI with or without ibrutinib for 1h at 37°C and then stimulated by the addition of 0.1-100 ng/ml IL-10 (PeproTech, Rocky Hill, NJ, USA). For viability assays, we calculated the mean relative viabilities to account for variability in spontaneous apoptosis rates in different patients' samples. We define the mean relative viability as the mean CLL cell viability of a particular sample (treated with ibrutinib in the presence or absence of NLCs at a certain time point), divided by the mean cell viability of the same sample at the same time point of control CLL cells cultured in suspension culture [49].

### FITC-DEXTRAN uptake and confocal microscopy of NLCs

For confocal microscopy experiments with NLCs, PBMCs from CLL patients were plated on glass coverslips in 24-wells plate in complete medium to generate NLCs as indicated. After 10 days, NLCs were treated with ibrutinib for 30 minutes and 1 hour, then the coverslips were transferred in new wells and incubated for 15 minutes at 37°C in PBS 5% FCS with 1 mg/ml of FITC-DEXTRAN (Sigma). Then, coverslips were fixed (4% paraformaldehyde for 10 minutes at RT), permeabilized (0.1% saponin for 20 minutes at RT), blocked with goat serum (30 min at 4°C) and incubated with anti-phalloidin-Alexa 568 (Invitrogen Life Technologies, 1:100, 1 hour at 4°C) followed by secondary antibody Goat anti-Rabbit-Alexa 594 (Invitrogen Life Technologies, 1:300, 1 hour at 4°C). Samples were counter-stained with DAPI and mounted in SlowFade Gold reagent (both from Invitrogen). Slides were analyzed using a TCS SP5 laser scanning confocal microscope equipped with 4 lasers (Leica Microsystems, Milan, Italy), images acquired with the LAS AF software and processed with Adobe Photoshop (Adobe Systems, San Jose, CA). Pixel intensity analyses were performed using the ImageJ (downloadable at http://rsbweb.nih.gov/ij/) and the LAS Application Suite (Leica Microsystems) softwares. Mean pixel intensity was calculated by defining a region of interest (ROI) and measuring green fluorescence pixel intensity. Results are expressed as fold change compared to untreated control [11].

### MTT assay and viability

NLCs activation was monitored using a yellow tetrazolium MTT assay (Trevigen, Gaithersburg, MD, USA). In this assay, dehydrogenases expressed by metabolically active cells convert MTT (3-[4,5-dimethylthiazol-2-yl]-2,5-dyphenyltetrazolium bromide) into intracellular purple formazan. NLC cells were cultured into a 96-well plate for 12 days and then treated with ibrutinib for 24 hours. Cells were then incubated with MTT at 37°C for 24 hours, followed by a 4h-incubation with 100 μL detergent reagent. Absorbance readings were performed at 570 nm in a microplate reader (Infinite M200, Tecan, Männedorf, Switzerland).

NLCs viability were tested by Annexin V-PI staining and analyzed by flow cytometry.

### Flow cytometry

To determine the abundance of NLCs surface markers, after 10 days the floating cells were removed by washes and the adherent cells were treated with ibrutinib for 1 hour or 24 hours, then detaching NLCs with PBS/EDTA solution. Cells were stained with the following antibodies and corresponding isotype controls: APC-conjugated CD14, CD163 (both BD Biosciences Pharmingen, San Jose, CA, USA), PE-conjugated CD11b CBRM 1/5 (the activated epitope of CD11b MAC-1) (eBioscience), pBTK Tyr^551^ (GeneTex, USA), pBTK Tyr^223^ (Novus Biological, Littleton CO, USA). Events were acquired using a FACSCalibur (Becton Dickinson, San José, CA, USA) or FACSAria cytometers and then analyzed by FlowJo Software (Tree Star, Ashland, OR, USA). In all the experiments, an isotype control sample for each condition was acquired to exclude autofluorescence background.

### Real time PCR

RNA was extracted with the RNeasy Plus Mini kit (Qiagen, Valencia, CA, USA). RNA (100ng) was reverse transcribed using Transcription High fidelity cDNA Synthesis kit (Roche Applied Science, Penzeberg, Germany). All samples were analyzed in real time on LightCycler 480v.2 (Roche) in duplicate. Amplification of the sequence of interest was normalized to an housekeeping reference gene (Glyceraldehyde 3-phosphate dehydrogenase, GAPDH) and compared to a calibrator sample (Universal Human Reference RNA; Stratagene, Cedar Creek, TX). Primers are listed in Supplementary Table S1.

### Immunoblotting

Proteins (80 μg/lane) were electrophoresed on SDS-polyacrylamide gradientgels (Biorad laboratories, Hercules, CA, USA). Membranes were immunoblotted with primary antibodies listed in Supplementary Table S2 and incubated with species-specific horseradish peroxidase (HRP)-conjugated secondary antibody (diluted 1:50000; GE Healthcare, Uppsala, Sweden) for 1 hour and developed using HRP conjugates WesternBright Sirius (Advasta, Menlo Park, CA, USA). Images were acquired and analyzed using Image Lab Software (Biorad Laboratories). After 10 days of culture, CLL cells were carefully removed by thoroughly pipetting, then NLCs were pretreated with ibrutinib for 1h following to stimulation with 100 ng/ml of LPS (Sigma-Aldrich) for 3 hours, 10 ng/ml of IL4 (Peprotech) for 30 minutes. CLL cells were stimulated with 1 ng/ml IL-10 after a pre-treatment of 1h with ibrutinib.

### Cytokine secretion assay (CSA)

To determine IL-10 secretion, NLCs were cultured for 10 days and then treated with ibrutinib for 24 hours and analyzed using CSA for IL-10 according to manufacturer's instructions (CSA Detection kit; Miltenyi Biotec). NLCs were immunostained with IL-10 catch reagent and incubated for 2 hours at 37°C to allow cytokine secretion. After washes, cells were labeled with IL-10 Detection antibody conjugated to PE and CD14 APC Ab. An isotype control sample for each condition was acquired to exclude autofluorescence background.

### Ex vivo flow cytometry analysis

Peripheral blood samples from CLL patients treated with ibrutinib were collected pre-treatment and after one month of treatment. PBMCs were stained with APC- conjugated CD14, CD206, CD11b (BD Biosciences) and Tie2 (R&D System). Events were acquired using a FACSAria cytometer and then analyzed by FlowJo Software.

### Statistical analysis

Data were analyzed using SPSS version 20.0 (SPSS, Chicago, IL, USA). In some experiments, results were normalized on control (100%) (vehicle-treated samples). Normalization was performed by dividing the value of a particular sample treated with ibrutinib to the value of the corresponding sample treated with vehicle DMSO. *P* values were calculated by Student t test (*P<0.05, **P<0.01). Data are presented as mean and standard error of the mean (SEM) is depicted as error bars.

## SUPPLEMENTARY MATERIALS FIGURE AND TABLES


