# Supplementary material for: Ibrutinib modifies the function of monocyte/macrophage population in chronic lymphocytic leukemia
Source: Oncotarget. 2016 Sep 1;7(40):65968–81. doi: 10.18632/oncotarget.11782 (PMC5323207; doi:10.18632/oncotarget.11782)
Supplement: Supplementary file 1 [file oncotarget-07-65968-s001.pdf]

# Ibrutinib modifies the function of monocyte/macrophage population in chronic lymphocytic leukemia

## SUPPLEMENTARY FIGURE AND TABLES

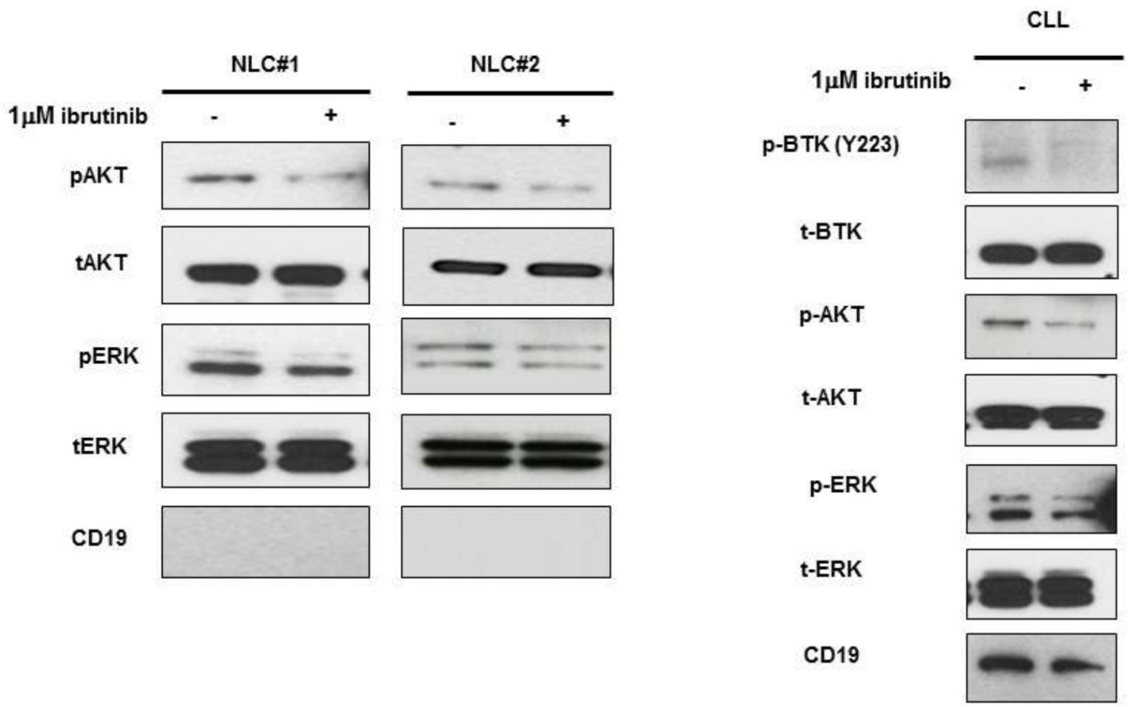


**Supplementary Figure S1: Ibrutinib impairs the activation of BTK-downstream signaling pathways.** Western Blot analysis of, AKT and ERK phosphorylation and total levels was performed after 1 hour of treatment with ibrutinib 1 µM. CD19 was used as a marker for CLL cells. On the right, western blot shows a representative CLL sample blotted for BTK, AKT, ERK and CD19.

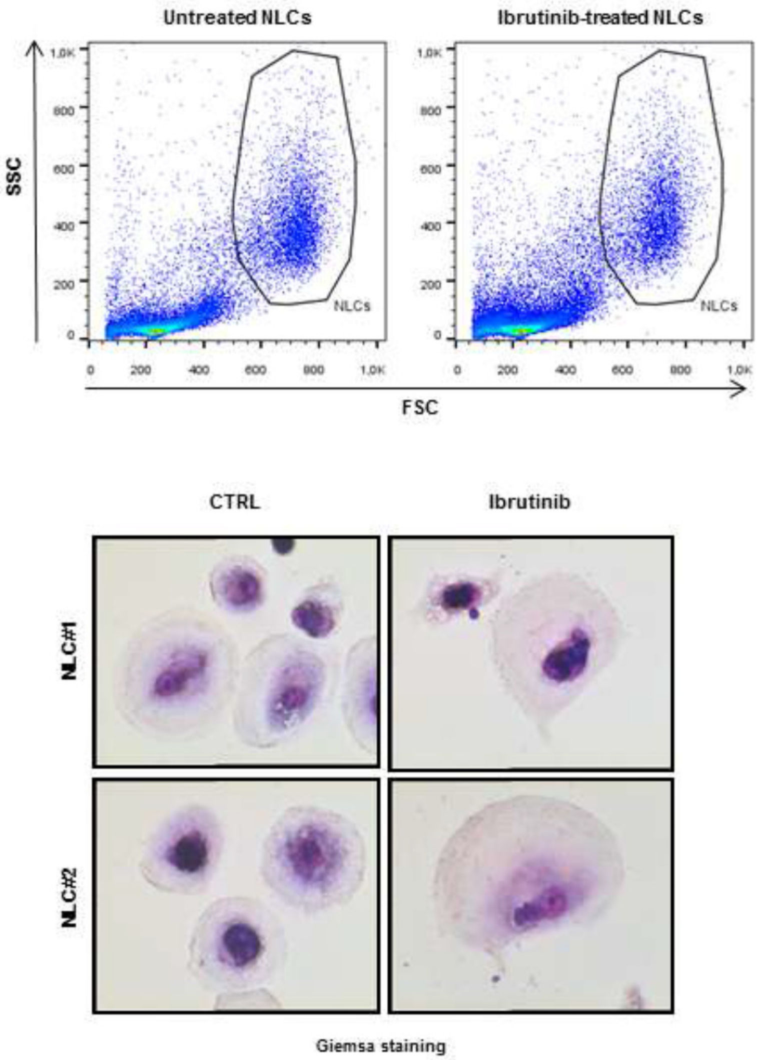


**Supplementary Figure S2: Ibrutinib does not affect NLCs formation.** Dot plots show the preserved morphology of NLCs after treatment with ibrutinib for 24 hours analyzed byflow-cytometric FCS and SSC plots. On the right, May-Grunwald Giemsa staining documented the morphology of NLCs in two representative samples.

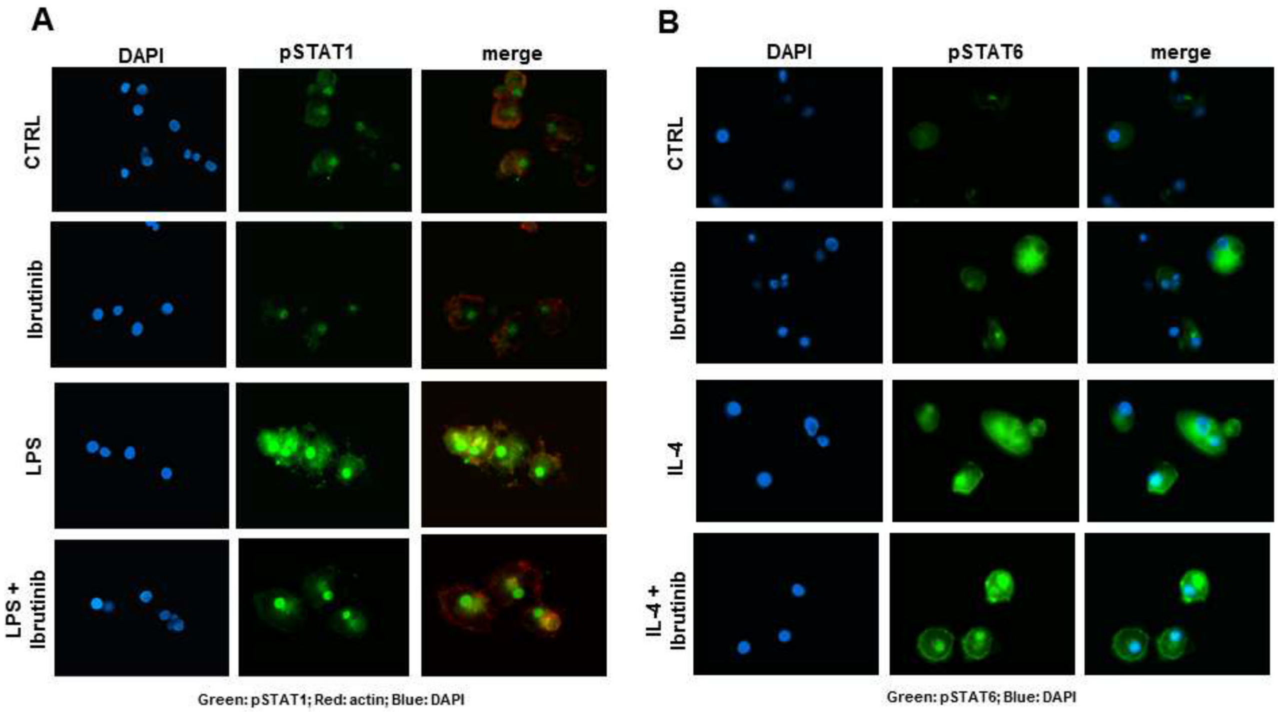


**Supplementary Figure S3: Ibrutinib stimulates M2 signaling pathways affecting M1 polarization in NLCs.** **A.** Immunofluorescence images show pSTAT1 expression after stimulation with LPS for 3 hours either after exposure or not with ibrutinib. NLCs were stained for pSTAT1, DAPI and phalloidin. **B.** Images show pSTAT6 expression after stimulation with IL-4 for 30 minutes either after exposure or not with ibrutinib. NLCs were stained for pSTAT6 and DAPI.

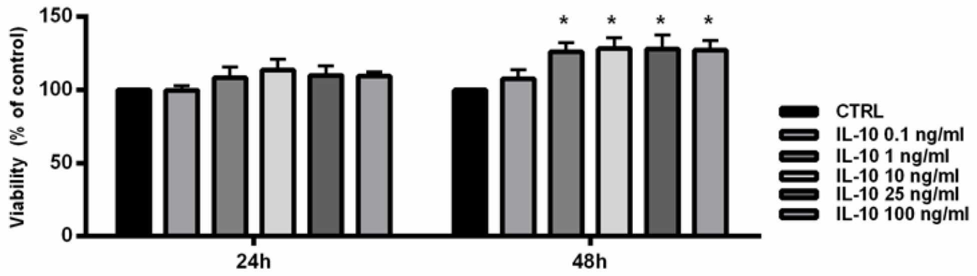


**Supplementary Figure S4: IL-10 mediates pro-survival signals in CD19+ CLL cells.** CD19+ CLL cells (n=4) were cultured for 2 days with a dose escalation of IL-10. Bar diagrams show CLL viability measured by Annexin V-PI staining (*P<0.05).

**Supplementary Table S1: Primers used in real-time PCR**

| Gene | Forward primer | Reverse primer |
|---|---|---|
| **CD163** | 5′-GAAGATGCTGGCGTGACAT-3′ | 5′-GCTGCCTCCACCTCTAAGTC-3′ |
| **IL10** | 5′-CATAAATTAGAGGTCTCCAAAATCG-3′ | 5′-AAGGGGCTGGGTCAGCTAT-3′ |
| **MRC1** | 5′-CACCATCGAGGAATTGGACT-3′ | 5′-ACAATTCGTCATTTGGCTCA-3′ |
| **CCL18** | 5′-ATGGCCCTCTGCTCCTGT-3′ | 5′-AATCTGCCAGGAGGTATAGACG-3′ |
| **IL1** | 5′-GGTTGAGTTTAAGCCAATCCA-3′ | 5′-TGCTGACCTAGGCTTGATGA-3′ |
| **TNFA** | 5′-CAGCCTCTTCTCCTTCCTGAT-3′ | 5′-GCCAGAGGGCTGATTAGAGA-3′ |

Commercially available primers (TaqMan Gene Expression Assays; Life Technologies) used were: Hs00237184_m1 (NAMPT) and Hs01125301_m1 (PD-L1).

**Supplementary Table S2: Antibodies used in immunoblotting**

| Antibody | Manufacturer |
|---|---|
| **Anti-phospho-BTK Tyr$^{551}$ rabbit** | GeneTex, Irvine, CA |
| **Anti-phospho-BTK Tyr$^{223}$ rabbit** | Cell Signaling Tech, Beverly, MA |
| **Anti-phospho-STAT1 mouse** | ECM Biosciences, Versailles, KY |
| **Anti-STAT1 mouse** | Cell Signaling Tech, Beverly, MA |
| **Anti-phospho-Erk 1/2 rabbit** | Cell Signaling Tech, Beverly, MA |
| **Anti-Erk 1/2 rabbit** | Cell Signaling Tech, Beverly, MA |
| **Anti-phospho-Akt rabbit** | Cell Signaling Tech, Beverly, MA |
| **Anti-Akt mouse** | Cell Signaling Tech, Beverly, MA |
| **Anti-phospho-IκBα mouse** | Cell Signaling Tech, Beverly, MA |
| **Anti-IκBα mouse** | Cell Signaling Tech, Beverly, MA |
| **Anti-phospho-STAT6 rabbit** | Cell Signaling Tech, Beverly, MA |
| **Anti-STAT6 rabbit** | Cell Signaling Tech, Beverly, MA |
| **Anti-SHIP1 rabbit** | Cell Signaling Tech, Beverly, MA |
| **Anti-phospho-STAT3 rabbit** | Cell Signaling Tech, Beverly, MA |
| **Anti-STAT3 mouse** | Cell Signaling Tech, Beverly, MA |
| **Anti-βactin mouse** | Abcam, Cambridge, UK |
